# Functional Relevance of the Interaction between Human Cyclins and the Cytomegalovirus-Encoded CDK-Like Protein Kinase pUL97

**DOI:** 10.3390/v13071248

**Published:** 2021-06-27

**Authors:** Martin Schütz, Mirjam Steingruber, Eileen Socher, Regina Müller, Sabrina Wagner, Merle Kögel, Heinrich Sticht, Manfred Marschall

**Affiliations:** 1 Institute of Clinical and Molecular Virology, Friedrich-Alexander University of Erlangen-Nürnberg (FAU), 91054 Erlangen, Germany; martin.schuetz@uk-erlangen.de (M.S.); mirjam.steingruber@fau.de (M.S.); mueller.regina@uk-erlangen.de (R.M.); sabrina.wagner@uk-erlangen.de (S.W.); merle.koegel@fau.de (M.K.); 2 Division of Bioinformatics, Institute of Biochemistry, Friedrich-Alexander University of Erlangen-Nürnberg (FAU), 91054 Erlangen, Germany; eileen.socher@fau.de (E.S.); heinrich.sticht@fau.de (H.S.); 3 Functional and Clinical Anatomy, Institute of Anatomy, Friedrich-Alexander University of Erlangen-Nürnberg (FAU), 91054 Erlangen, Germany

**Keywords:** human cytomegalovirus, virus-encoded kinase pUL97, viral CDK ortholog, interaction with human cyclin T1, replication-impaired viral mutants, defects in pUL97 activities, pUL97–cyclin functional importance

## Abstract

The replication of human cytomegalovirus (HCMV) is characterized by a complex network of virus–host interaction. This involves the regulatory viral protein kinase pUL97, which represents a viral cyclin-dependent kinase ortholog (vCDK) combining typical structural and functional features of host CDKs. Notably, pUL97 interacts with the three human cyclin types T1, H and B1, whereby the binding region of cyclin T1 and the region conferring oligomerization of pUL97 were both assigned to amino acids 231–280. Here, we addressed the question of whether recombinant HCMVs harboring deletions in this region were impaired in cyclin interaction, kinase functionality or viral replication. To this end, recombinant HCMVs were generated by traceless BACmid mutagenesis and were phenotypically characterized using a methodological platform based on qPCR, coimmunoprecipitation, in vitro kinase assay (IVKA), Phos-tag Western blot and confocal imaging analysis. Combined data illustrate the following: (i) infection kinetics of all three recombinant HCMVs, i.e., ORF-UL97 ∆231–255, ∆256–280 and ∆231–280, showed impaired replication efficiency compared to the wild type, amongst which the largest deletion exhibited the most pronounced defect; (ii) specifically, this mutant ∆231–280 showed a loss of interaction with cyclin T1, as demonstrated by CoIP and confocal imaging; (iii) IVKA and Phos-tag analyses revealed strongly affected kinase activity for ∆231–280, with strong impairment of both autophosphorylation and substrate phosphorylation, but less pronounced impairments for ∆231–255 and ∆256–280; and (iv) a bioinformatic assessment of the pUL97–cyclin T1 complex led to the refinement of our current binding model. Thus, the results provide initial evidence for the functional importance of the pUL97–cyclin interaction concerning kinase activity and viral replication fitness.

## 1. Introduction

Human cytomegalovirus (HCMV) is a worldwide distributed opportunistic pathogen of the β-*Herpesvirinae* subfamily that establishes lifelong latent infection in humans. In the immunocompetent host, HCMV may remain asymptomatic, whereas in immunosuppressed individuals, such as transplant recipients, tumor and AIDS patients, HCMV infection can lead to severe symptoms and a life-threatening viral pathogenesis [1,2]. Most seriously, congenital HCMV infection represents a considerable risk for the unborn child to obtain developmental defects or cytomegalovirus inclusion disease [3,4]. Viral pathogenesis is closely linked to the efficiency of viral replication in individual tissues, whereby the pathogenic determinants based on virus–host interaction are insufficiently understood. On the molecular level, recent investigations stressed the importance of multiprotein complexes consisting of viral and host components [5,6,7,8,9]. Notably, HCMV replication drastically interferes with cell cycle regulation, a process in which the HCMV-encoded protein kinase pUL97 massively phosphorylates the checkpoint regulator retinoblastoma protein (Rb) [10,11,12]. This initial Rb inactivation, followed by further viral regulatory steps of intervention, ultimately results in an early S phase cell cycle arrest accompanied by the massive dysregulation of cyclin-dependent kinases (CDKs) and cyclins termed pseudomitosis [1,13,14,15]. Interestingly, HCMV encodes the serine-threonine protein kinase pUL97 that represents a viral CDK ortholog (vCDK) as it combines typical structural and functional features of host CDKs [16]. Expressed with early–late kinetics, pUL97 is primarily a nuclear kinase, possessing a variety of phosphorylation activities including autophosphorylation, is later transported to cytoplasmic viral replication compartments (cVACs) and ultimately packaged into virions as a tegument protein. In addition to autophosphorylation, pUL97 mediates the phosphorylation of other viral tegument proteins, such as pp65/pUL83 and pUL69 [17,18,19,20,21]. A pronounced complexity of pUL97 protein interactions and substrate phosphorylation has been described [16], as illustrated by the number of viral and cellular target proteins identified so far [22,23,24]. The functionality of these substrates spans various regulatory aspects of viral replication, such as nuclear egress, intrinsic immunity, genome replication and gene expression [16,25,26]. Previously, we identified a specific feature of pUL97, in that it associates with human cyclins of the three types T1, H and B1 [9,27,28,29,30]. These three cyclins obviously possess different affinities in pUL97 binding [29] and might also play different functional roles [9]. Based on these observations, pUL97 is considered a multiple cyclin-binding kinase, and, in addition to the types T1, B1 and H, further cyclin binding activities, e.g., cyclin A, might also play another interesting role [16,28,31].

In general terms, cyclins are a large family with 29 proteins in humans, structurally defined by the presence of the so-called cyclin box, a domain of approximately 100 amino acid residues that forms α-helices and provides an interface for binding to CDKs. Their intracellular concentrations vary in a cyclical fashion during the cell cycle and these oscillations, specifically the fluctuations in the gene expression of cyclins, then induce oscillations in CDK activity driving the cell cycle [32]. Of note, the cell-cycle-related CDKs are typically characterized by multiple cyclin binding, whereas transcriptional CDKs are mostly single cyclin binding. The CDK–cyclin complex formation results in activation of the CDK active site, and complete activation requires distinct regulatory steps of site-specific phosphorylation. Cyclin subfamilies comprise the three major groups, i.e., the cyclin B group (including B1), cyclin C group (including H, T1) and cyclin Y group. While cyclin B1 represents the activating subunit of cell-cycle-related CDKs 1, 2 and 3, the cyclins H and T1 are typically associated with the transcriptional CDKs 7 or 9, respectively [32,33].

The interaction pUL97–cyclin B1 was found to be phosphorylation-dependent for both proteins, and cyclin B1 (but not H) was phosphorylated by pUL97 in vitro [9]. It was speculated whether cyclin B1 binding and modulation through pUL97 might be linked to the HCMV-induced alteration of the cell cycle and host CDK–cyclin machinery, but this awaits further experimental confirmation. The role of cyclin types T1 and H, on the other hand, has been assigned to a bridging function of pUL97–pUL97 dimerization/oligomerization [16]. Importantly, the minimal binding regions responsible for pUL97–cyclin T1 interaction and pUL97–pUL97 oligomerization showed a complete overlap in N-terminal amino acids 231–280 [9,28,34]. These data point to the concept that cyclin T1 binding (similarly to the related cyclin H) may be directly involved in pUL97 oligomerization, thereby putatively contributing to the full catalytic activity of pUL97. On the basis of combined findings, our latest model attributed the activated state of the pUL97 kinase to distinct regulatory factors, including cyclin binding, pUL97 oligomerization, autophosphorylation and complex-specific coregulatory events. Examples for the latter aspect were found in the binding of pUL97 to its substrate pp65, which is based on a cyclin bridging function, as determined with a pp65 mutant lacking its cyclin-docking motif [35]. Importantly, a functional link between pUL97 autophosphorylation, oligomerization and a fully activated catalytic state has been derived from data published earlier [9,28,34]. Moreover, the ternary complexes identified for pUL97–cyclin-H–CDK7 in vitro, which may exert multiple phosphorylation activities, are an example of multifactorial regulation [9]. However, a central question remains elusive so far—namely, whether the functional relevance of cyclin binding may also be measurable in the context of viral replication. To this end, recombinant HCMVs were generated that harbor genomic deletions in region 231–280 of ORF-UL97, and experimentation was performed to address a putative impairment of cyclin interaction, kinase activity or viral replication. The results provide initial evidence for the functional importance of the pUL97–cyclin interaction that drives the efficiency of viral replication, and a refined concept of the regulatory mode of cyclin binding is discussed.

## 2. Materials and Methods

### 2.1. Cell Culture, Virus Infection and Transient Transfection

Primary human foreskin fibroblasts (HFFs, own repository of primary cell cultures) were cultivated in minimal essential medium (MEM) containing 10% FCS, 1× GlutaMAX^TM^ (35050038, Thermo Fisher Scientific, Waltham, MA, USA) and 10 μg/mL gentamicin (22185.03, SERVA, Heidelberg, Germany). Parental or recombinant HCMVs derived from strain AD169 were used for infection experiments at a multiplicity of infection (MOI) of 1 or lower, as indicated for specific experiments. After incubation for 90 min at 37 °C, virus inocula were removed and replaced with fresh growth medium. Transient transfection of expression plasmids was performed as described before [36].

### 2.2. Generation of Recombinant HCMV

BAC HB5/AD169-GFP [37,38] was used for the generation of HCMV UL97 deletion mutants ∆231–255, ∆256–280 and ∆231–280. Deletions were achieved by a two-step markerless Red recombination system as described previously [39,40]. The following primers were used:

UL97∆231–280 for 5′-ggacgcggtgctcgaagaaaacgacgtggagctgcgcgcgaccacgtccatccgcggcctTAGGGATAACAGGGTAATCGATTT-3′;
UL97∆231–280 rev 5′-acatacgcgggtcgcacgtaaggccgcggatggacgtggtcgcgcgcagctccacgtcgtGCCAGTGTTACAACCAATTAACC-3′;
UL97∆256–280 for 5′-ccgcattccgcagccgctcagcggtagttccggggaggaaaccacgtccatccgcggcctTAGGGATAACAGGGTAATCGATTT-3′;
UL97∆256–280 rev 5′-acatacgcgggtcgcacgtaaggccgcggatggacgtggtttcctccccggaactaccgcGCCAGTGTTACAACCAATTAACC-3′;
UL97∆231–255 for 5′-ggacgcggtgctcgaagaaaacgacgtggagctgcgcgcgtccgccacggcggtggaggcTAGGGATAACAGGGTAATCGATTT-3′;
UL97∆231–255 rev 5′-cgtcgtgtgacgtggagtcggcctccaccgccgtggcggacgcgcgcagctccacgtcgtGCCAGTGTTACAACCAATTAACC-3′.

Briefly, primers spanning sequences complementary to both up- and downstream regions of the deletion target sequence were used to amplify a kanamycin-conferring resistance cassette. Homologous recombination of the amplified cassette with the original sequence resulted in deletion of the desired sequence and replacement by the kanamycin resistance cassette. Kanamycin-resistant clones were then selected and correct recombination was verified by nucleotide sequencing. In the next step, the kanamycin cassette was removed through the activity of arabinose-induced expression of the restriction enzyme *Sce*I and a subsequent second round of recombination that joined the flanking UL97-specific sequences. Recombinant viruses were reconstituted by transfection of the generated BACmids into HFFs using the Fugene transfection reagent (Promega, Madison, WI, USA). The correctness of gene sequences and deletions was verified by repeated sequence analyses of PCR fragments derived from both templates of BACmid DNA as well as reconstituted virion DNA.

### 2.3. Antibodies

Antibodies used in this study were mAb-UL97.01, mAb-UL50.01 (kindly provided by Tihana Lenac Rovis, University of Rijeka, Rijeka, Croatia), mAb-UL44 (kindly provided by Bodo Plachter, University of Mainz, Mainz, Germany), mAb-β-Actin (A5441, Sigma-Aldrich, St. Louis, MO, USA), mAb-UL69 [41], mAb-pp65 (kindly provided by William Britt, University of Alabama, Birmingham, AL, USA), mAb-Rb (4H1, Cell Signaling, Danvers, MA, USA #9309), mAb-Lamin A/C (EPR4100, ab108595, Abcam, Cambridge, UK), mAb-Cyclin B1 (sc-245, Santa Cruz Biotechnology, Dallas, TX, USA), pAb-Cyclin B1 (AF6000, R&D Systems), pAb-Cyclin T1 (ab226851, Abcam, Cambridge, UK), pAb-Cyclin H (LS-C331195, Lifespan Biosciences, Seattle, WA, USA), pAb-TPE (ab133473, Abcam, Cambridge, UK), pAb-Rb pSer807/811 (#9308, Cell Signaling, Danvers, MA, USA), pAb-Rb pThr373 (ab52975, Abcam, Cambridge, UK), pAb-Rb pSer608 (#2181, Cell Signaling, Danvers, MA, USA), pAb-Rb pSer796 (#9301, Cell Signaling, Danvers, MA, USA), pAb-Cyclin H pThr315 (GTX55316, GeneTex, Irvine, CA, USA), pAb-lamin A/C pSer22 (ABIN1532183, Antibodies online, Aachen, Germany), anti-mouse Alexa 350 (A-11045, Thermo Fisher Scientific, Waltham, MA, USA), anti-rabbit Alexa 555 (A-21422, Thermo Fisher Scientific, Waltham, MA, USA), anti-goat Alexa 555 (A-21432, Thermo Fisher Scientific, Waltham, MA, USA), anti-mouse Alexa 647 (A-28181, Thermo Fisher Scientific, Waltham, MA, USA) and anti-rabbit Alexa 647 (A-21245, Thermo Fisher Scientific, Waltham, MA, USA).

### 2.4. Coimmunoprecipitation (CoIP), In Vitro Kinase Assay (IVKA), Phosphate Affinity (Phos-tag) SDS-PAGE and Western Blot (WB) Analyses

For the investigation of expression patterns, HCMV-infected HFFs were harvested and lysed between 3 and 5 days post-infection (d.p.i.), when approximately 80% of cells showed a visible cytopathic effect (CPE). These conditions of CPE-based sample collection were chosen to ensure the consistent detectability of pUL97 for the CoIP settings and best possible conclusions regarding pUL97–cyclin interaction. The coimmunoprecipitation assay (CoIP) was performed as described previously [29]. Immunoprecipitated samples (IP) were subjected to standard SDS-PAGE/Wb procedures [42,43]. Alternatively, IP samples could be subjected to an in vitro kinase assay (IVKA) to assess the kinase activity of pUL97. To this end, the IP samples were washed twice in 500 µL HNTG buffer (50 mM HEPES (pH 7.5), 150 mM NaCl, 1 mM EDTA, 10% glycerin and 0.1% Triton X-100) and two times in 500 µL kinase basis buffer (20 mM Tris/Cl (pH 7.5), 0.5 mM MnCl_2_). Next, IP samples were incubated for 60 min under vigorous shaking (1400 rpm/min) at 30 °C with 30 µL kinase assay buffer (basis buffer supplemented with 1 µM ATP and 200 ng ATPγS). As specificity control, a sample was simultaneously incubated in kinase assay buffer without the addition of ATPγS. ATPγS was used as the thiophosphorylation donor for in vitro conversion of the kinase substrates. Next, 3 µL of 25 mM p-nitrobenzyl mesylate (PNBM, Abcam, Cambridge, UK) was added to the reaction to alkylate thiophosphates and samples were incubated for 30 min at 25 °C. The reaction was stopped by denaturation at 94 °C for 10 min with 30 µL of 2x-SDS-PAGE loading buffer. After standard SDS-PAGE, phosphorylated proteins were detected via thiophosphate ester (TPE)-specific antibody stainings on Wbs. To confirm phosphorylated proteins, Wbs were restained using protein-specific antibodies. Additionally, Phos-tag gels were used for the identification of phosphorylated protein varieties. For this purpose, HCMV-infected HFFs were harvested and lysed in CoIP buffer without EDTA for 20 min on ice. Half volumes of the samples were subjected to lambda phosphatase (λ-PP) treatment by supplementing lysates with 1xPMP buffer, 1 mM MnCl_2_ and 0.5 μL of lambda phosphatase (P0753S, New England Biolabs, Ipswich, MA, USA). As a control, identical volumes of CoIP buffer were added to the second half of samples, which were then incubated at 30 °C for 45 min and subsequently denatured at 94 °C for 10 min. Finally, all samples were analyzed by SDS-PAGE, supplemented with Phos-tag reagent [44] (AAL-107, Wako PureChemical Industries, Osaka, Japan) according to manufacturer’s instructions, and subjected to subsequent Wb immunostaining.

### 2.5. Indirect Immunofluorescence Assay and Confocal Laser Scanning Microscopy

HFFs were seeded on coverslips for infection. At 3 d.p.i., cells were fixed and permeabilized as described before [45,46,47]. Images were acquired using a TCS SP5 confocal laser scanning microscope (Leica Microsystems, Wetzlar, Germany). Visual microscope counting was performed for quantitative evaluations. In our previous studies [27,28], the colocalization of cyclins with pUL97 as well as its substrate proteins pUL69 and pUL44 has been investigated in several different types of HCMV-permissive cells and did not show basic differences; therefore, in the present study, the mutant analysis was focused on HFFs that produce the most distinct patterns of microscopically detectable cyclin colocalization [9,30].

### 2.6. Quantitative Polymerase Chain Reaction (qPCR)

Infection experiments were performed at MOI of 0.01 or 0.001 using parental HCMV WT compared to UL97 mutants. Viral replication kinetics were analyzed by quantitative real-time PCR (qPCR) as described previously [48]. Briefly, viral supernatants were harvested at indicated time points and extracellular viral genome equivalents were determined by the amplification of a specific region in the IE1 gene locus (ORF-UL123, exon 4) using a FAM/TAMRA-labeled probe. Additionally, cells were harvested at indicated time points, DNA was extracted using Quick-DNA MicroProp Kit (Zymo Research, Freiburg, Germany) and intracellular viral genome equivalents were determined. Albumin genome equivalents were measured in parallel as a housekeeping gene using specific primers and an albumin FAM/TAMRA probe. Subsequently, HCMV genome equivalents were normalized to albumin signals. Each value given is a mean of quadruplicates ± SD.

### 2.7. HCMV GFP-Based Replication Assay

HCMV GFP-based replication assays were performed as described previously [49,50]. In brief, 200,000 HFFs were cultivated in 12-well plates for the infection with parental HCMV AD169-GFP or UL97 deletion mutants ∆231–255, ∆256–280 and ∆231–280 at a MOI of 0.25 GFP-FU/cell (i.e., 25% GFP-forming doses of a multi-round infection measured at 7 d) and treated with maribavir (MBV) or ganciclovir (GCV) at serial concentrations. At 7 d.p.i., the cells were lysed and total lysates were subjected to automated GFP quantitation using a Victor 1420 multilabel counter (Perkin-Elmer, Waltham, MA, USA). Mean values ± SD of quadruplicate determinations are given (infections in duplicates, GFP measurements in duplicate).

### 2.8. Bioinformatic Methods

Structural analysis of the cyclin T1 interfaces was performed based on the complex crystal structures with Tat [51,52] and CDK9 [52]. Structure presentations were generated with Chimera 1.15 [53].

## 3. Results

### 3.1. Construction of Expression Plasmids and Recombinant Viruses Encoding Mutant Versions of ORF-UL97

**Figure 1:**
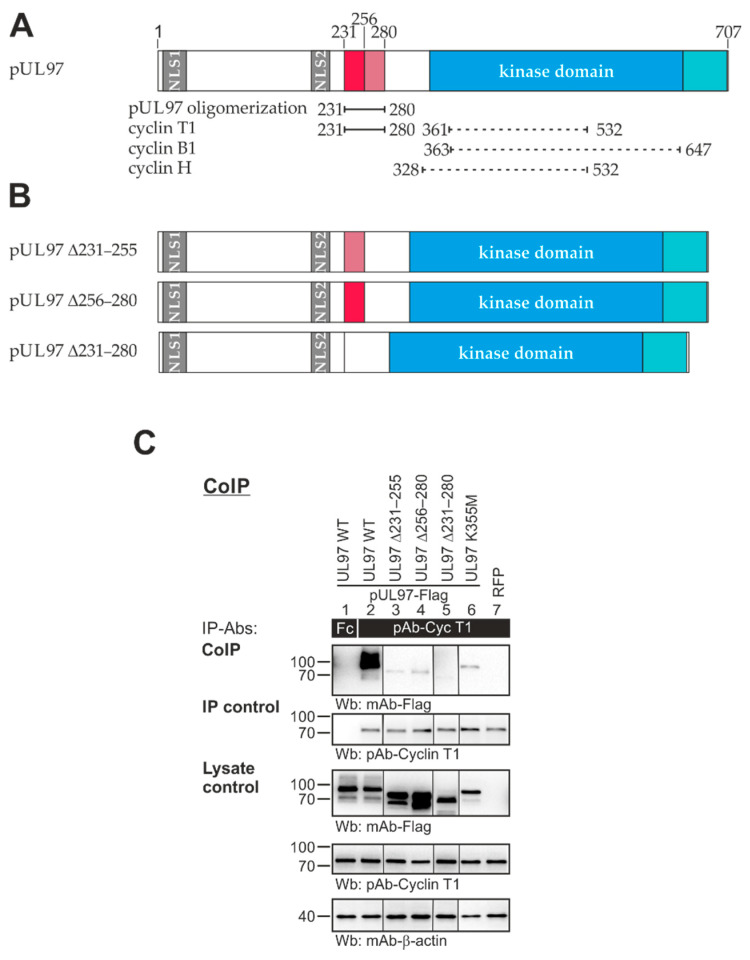
Schematic view of ORF-UL97 constructs used in this study. (**A**) Wild-type pUL97 and currently identified binding regions for pUL97 oligomerization as well as binding to human cyclin T1, B1 and H are depicted. The N-terminus contains two nuclear localization signals (NLS). Oligomerization of pUL97 and binding to cyclin T1 is determined by amino acid region 231–280. Recombinant viruses were generated harboring deletions within this region (red shading). Modeling of in silico binding interfaces predicted extended interfaces for cyclin T1, B1 and H (dashed lines). The catalytically active kinase domain is, based on sequence homologies, located between amino acids 337 and 651 (blue). This domain can be extended to 706 based on biochemical validation (turquoise). (**B**) Recombinant viruses generated for this study. A Red recombination system was used to generate deletion mutants UL97 ∆231–255, ∆256–280 and ∆231–280 from the parental BACmid HB5/AD169-GFP. (**C**) 293T cells were transiently transfected with plasmids coding for pUL97-Flag WT or mutant versions (K355M is a catalytically inactive replacement mutant). Transfection with an RFP-coding plasmid was used as negative control. At 2 d post-transfection, cells were lysed and human cyclin T1 was immunoprecipitated using specific Abs; a rabbit Fc fragment served as specificity control. Subsequently, CoIP samples and lysate controls taken prior to the addition of IP-Abs were subjected to standard Wb analysis using specific Abs as indicated.

The rationale for the construction of recombinant HCMVs carrying mutant versions of ORF-UL97 was based on previous findings. Our mapping analyses using deletion mutants of pUL97 in transient expression systems pointed to the importance of the amino acid region 231–280 that is placed outside of the globular domain in the poorly structured N-terminus of pUL97 (Figure 1A). Deletion of this region suggested a hot spot of pUL97 protein interactions and relevance for kinase functionality (reviewed in [16]). The use of truncated versions of pUL97 in CoIP settings led to the definition of 231–280 as a core region responsible for cyclin T1 interaction [28] and at the same time as the minimal region referring pUL97 dimerization and oligomerization [34]. Moreover, the lack of region 231–280 in pUL97 resulted in a strongly reduced activity of pUL97 autophosphorylation [15,34], thus pointing to the basic importance of the overall pUL97 functionality. In order to illustrate this point in more detail, three different recombinant HCMVs were generated through markerless BACmid technology, resulting in the deletion mutants HCMV UL97 ∆231–255, ∆256–280 and ∆231–280 (Figure 1B). Since these deletion versions of pUL97 expressed by the mutant viruses did not contain tag sequences, the ability of pUL97–pUL97 oligomerization/self-interaction could not be reinvestigated using infected-cell material. However, the transient expression plasmids of these deletion versions, which represented the starting material for the generation of recombinant viruses, contained Flag- or HA-tags, respectively (see also [28,34]). Using these plasmids in transfected-cell-based CoIP settings, the complete lack of pUL97–pUL97 self-interaction as well as pUL97–cyclin T1 interaction (endogenous cyclin T1) could be confirmed for the largest deletion, ∆231–280, as well as partial loss of cyclin T1 binding for ∆231–255 and ∆256–280 (Figure 1C). The more detailed analyses of protein interaction and viral replication efficiency were then performed with the use of recombinant viruses, as described below.

### 3.2. Determination of Replication Kinetics of the Three Viral Deletion Mutants

**Figure 2:**
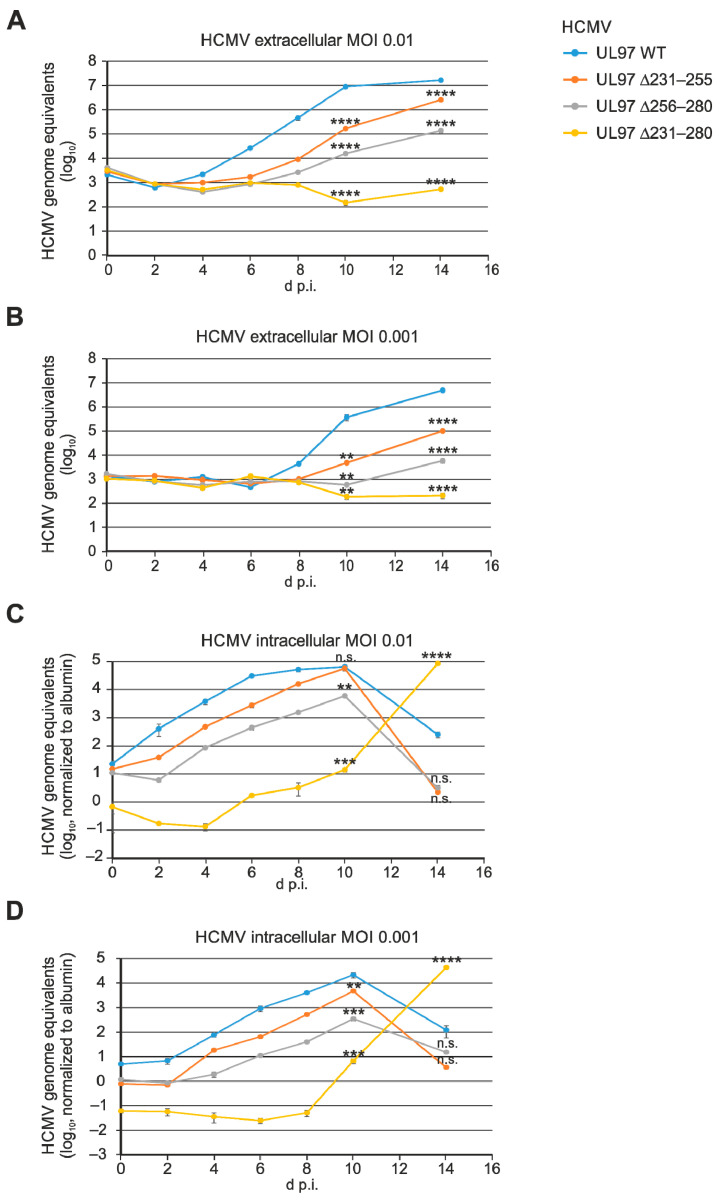
HCMV replication kinetics of the UL97 deletion mutants. HFFs were infected with parental HCMV WT or UL97 mutants at MOI of 0.01 (**A**,**C**), or 0.001 (**B**,**D**). Viral supernatants were harvested at indicated time points and viral genome equivalents were determined by qPCR (**A**,**B**). Alternatively, cells were harvested at indicated time points and intracellular viral genome equivalents were determined together with albumin as housekeeping gene via qPCR. Subsequently, HCMV genome equivalents were normalized to albumin signals (**C**,**D**). Each value is given as a mean of quadruplicates and standard errors of mean (SEM) are shown. Statistical analysis was performed using ordinary one-way ANOVA and Dunnett’s test compared to HCMV UL97 WT. ****, *p* < 0.0001; ***, *p* < 0.001; **, *p* < 0.01; n.s., not significant.

In order to address the question of whether the three small deletions in ORF-UL97 may have an impact on virus replication efficiency, a kinetic analysis was performed by the use of viral genome-specific qPCR. It should be noted that earlier work by other researchers and our group made clear that deletion of the entire ORF-UL97, or replacement by a catalytically inactive K355 mutant, did not lead to an entire replication defect, but to a severe impairment of replication efficiency of the rescue virus [20,54,55]. Here, we monitored the parallel time courses of infection between the parental ORF-UL97 wild-type (WT) virus and the deletion mutants HCMV UL97 ∆231–255, ∆256–280 and ∆231–280 in the period of 0–14 d.p.i. (Figure 2). Identical genome equivalents were used for infection performed with MOI of 0.01 or 0.0001 as indicated. Viral genomic load was determined by qPCR using either the medium supernatants of infected cells (Figure 2A,B) or cellular lysates (Figure 2C,D). In all cases, the deletion mutant UL97 ∆231–280 showed strong replication retardation and quantitative impairment compared to WT, as expressed by a reduction of approximately 4 log-range in the supernatants at 14 d.p.i., which was similarly seen at both MOIs (Figure 2A,B). The smaller deletion mutants, ∆231–255 and ∆256–280, showed an intermediate phenotype with a less severe replicative impairment. Interestingly, the qPCR kinetics measured for the cellular lysates indicated a very similar effect for the two intermediate mutants, but a punctually different picture for UL97 ∆231–280. The latter was characterized by a most pronounced retardation of an increase in intracellular viral genomic load, but at later time points, after 10 d.p.i., there was a strong accumulation of viral load in the cells. This may be explained by the reduced cytopathic effect (CPE) of this mutant, UL97 ∆231–280, which led to the accumulation of viral genomes, instead of the degradation in CPE-positive cells seen for the other two mutants and WT (Figure 2C,D). In addition, it should be mentioned that, as far as the detection of release of infectious virus is concerned, stocks of infectious supernatant virus could be reliably produced for all mutants, and these supernatant stock viruses could be used for infection experiments in the quantitative HCMV GFP-based replication assay (for comparison, see Figure 6) and for the semi-quantitative determination of virus-induced CPEs by visual microscopic inspection. The results of these confirmatory experiments were strongly consistent with the qPCR-based HCMV replication kinetics shown in this study (Figure 2). In panels C,D, the qPCR data indicate that viral genomic DNA is already detectable intracellularly at 0 d.p.i.. There is an initial decrease in viral copy numbers for the ∆231–280 mutant, which might indicate some impairment of viral entry. Combined, these findings led to the conclusion that deletion of the region UL97 ∆231–280 effected a pronounced replicative defect, whereas the partial deletions ∆231–255 and ∆256–280 had a lower impact on viral replication competence. The functional basis of these viral phenotypes was then addressed by the characterization of pUL97-specific regulatory features.

### 3.3. Determination of the Phenotypes of pUL97–Cyclin Interaction for Mutant Viruses

**Figure 3:**
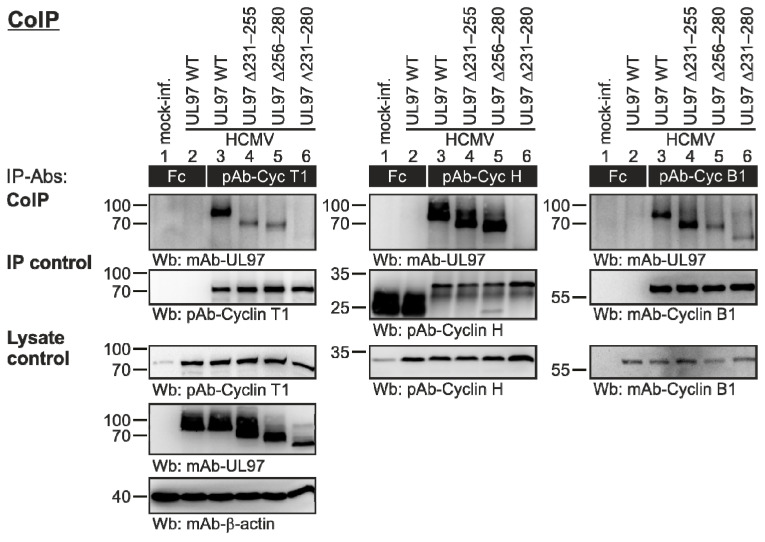
Interaction analysis of pUL97 with human cyclins. HFFs were infected with HCMV WT or UL97 mutant versions at an MOI of 1. At 4 d.p.i. (WT) or the time point referring to approximately 80% of virus-induced CPE (mutants), cells were lysed and human cyclins T1, H and B1 were immunoprecipitated using specific Abs; a rabbit Fc fragment served as specificity control. Subsequently, CoIP sample and lysate control taken prior to the addition of IP-Abs were subjected to standard Wb analysis using specific Abs as indicated. Note that pUL97 ∆231–280 no longer interacted with cyclin T1 and H, whereas all mutants retained the interaction with cyclin B1.

In the first step, the pattern of pUL97 interaction with cellular cyclins was analyzed by CoIP experiments. To this end, HCMV-infected cells were lysed at 4 d.p.i. and subjected to cyclin-specific CoIP using pAb-cyclin T1, pAb-cyclin H or pAb-cyclin B1, respectively (Figure 3). Lysate control stainings indicated that the level of pUL97 expressed by the ∆231–280 recombinant virus was lower than WT, so that the protein may have some reduced stability due to the short deletion. As expected from the previous work, HCMV UL97 ∆231–280 showed a lack of cyclin T1 interaction (Figure 3, left upper panel, lane 6, compare to WT in lane 3), and both partial deletion mutants, ∆231–255 and ∆256–280, showed also here an intermediate phenotype with reduced cyclin T1 interaction (Figure 3, lanes 4–5). Moreover, mutant UL ∆231–280 also showed a defect in the interaction with the cyclin H (Figure 3, central upper panel, lane 6), while ∆231–255 and ∆256–280 showed preservation of the WT-like, strong cyclin H interaction. In contrast, cyclin B1 interaction was positive in all cases, whereby ∆231–280, and also ∆256–280, produced reduced CoIP signal intensity compared to WT and mutant ∆231–255. The CoIP results are considered in line with the replication kinetics, in that the largest deletion showed the most pronounced impairment of cyclin binding activity, while the two smaller deletions were found to be intermediate. Combined, these data strongly argue for a binding defect of pUL97 expressed by HCMV UL97 ∆231–280 for the functionally related cyclins T1 and H, but not for cyclin B1, or only to a lesser extent.

### 3.4. Characterization of the pUL97 Catalytic Properties in Terms of Auto- and Substrate Phosphorylation

**Figure 4:**
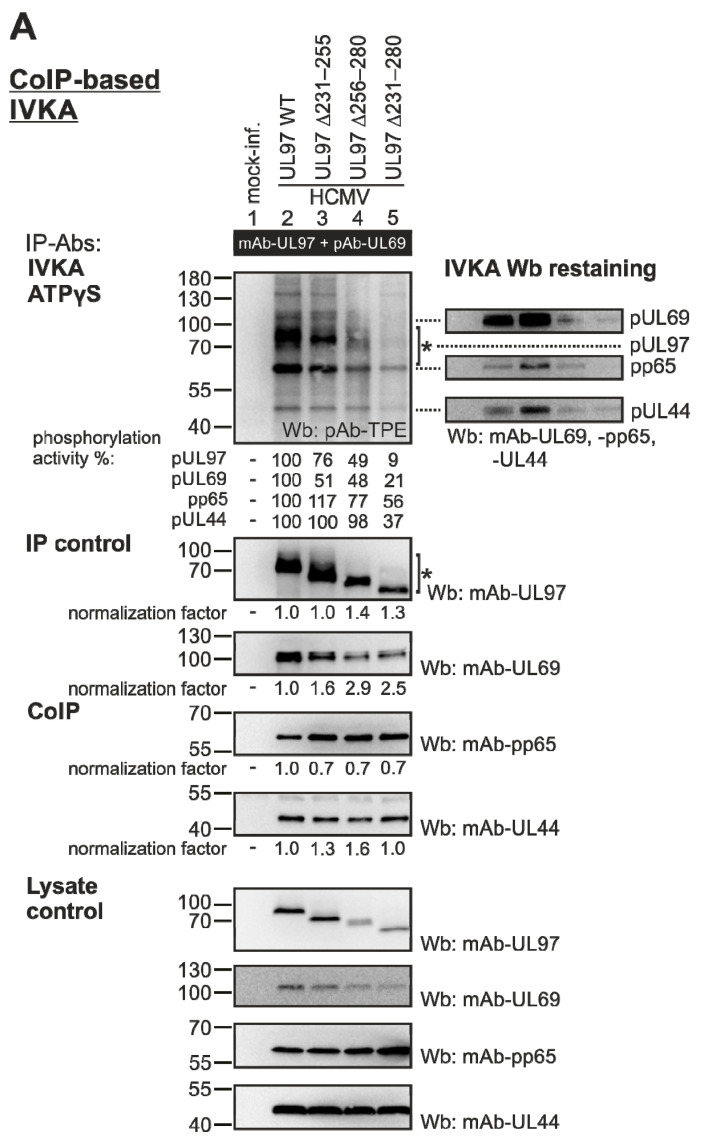

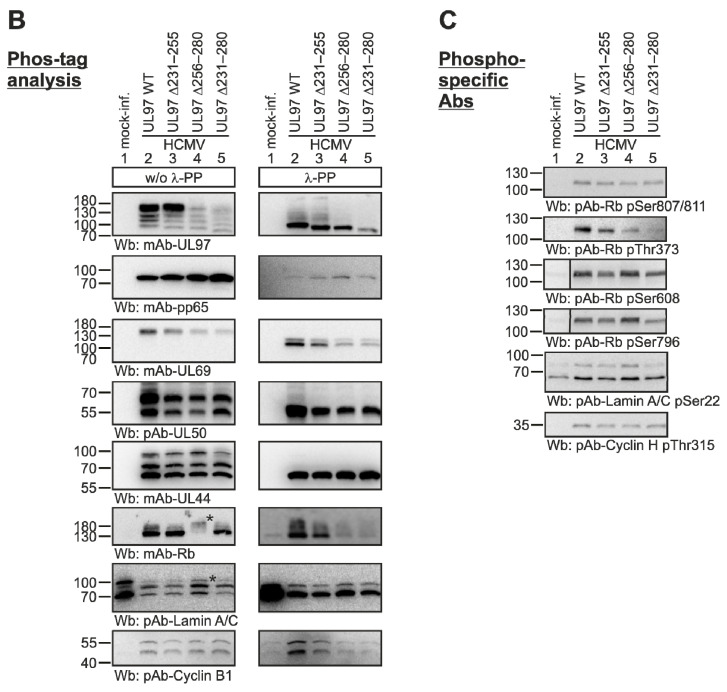
Measurement of viral pUL97-specific kinase activities, using (**A**) in vitro kinase assay, (**B**) Phos-tag analysis and (**C**) phosphorylation-specific antibodies. HFFs were infected with HCMV WT or UL97 mutant versions at an MOI of 1. At 4 d.p.i. (WT) or the time point referring to approximately 80% of virus-induced CPE (mutants), cells were lysed and kinase activity of pUL97 was analyzed by in vitro kinase assay (IVKA) (**A**). pUL97 was immunoprecipitated together with pUL69 using specific antibodies (Abs). Lysate control was taken prior to CoIP. CoIP-enriched proteins were subjected to an IVKA reaction utilizing ATPγS, which is used by kinases to thiophosphorylate substrates. Subsequent alkylation of thio-phosphorylated substrates with p-nitrobenzyl mesylate yields thiophosphate esters (TPE), which were detected via specific Abs on Western blot (Wb). To confirm phosphorylated proteins, Wbs were restained using protein-specific Abs. Alternatively, phosphorylation pattern of relevant pUL97 substrates was analyzed via Phos-tag SDS-PAGE (**B**) or phospho-specific Abs (**C**). For details of the methodological procedures of the use of Phos-tag and phospho-specific Abs, see Section 2.4. *, Note the occurrence of an unusual phospho-specific band for Rb and a so far unexplained upregulation effect for nuclear lamin A/C, respectively.

In order to analyze the properties of pUL97-mediated auto- and substrate phosphorylation, a CoIP-based in vitro kinase assay (IVKA) was performed. To this end, HFFs were infected with the parental HCMV WT or one of the three UL97 deletion mutants. Total cell lysates were subjected to IP using antibodies against pUL97 and pUL69, representing one of the major pUL97-specific substrates. Two other viral substrates, pp65 and pUL44, were detected as additional CoIP targets. The quantities of intracellular expression of pUL97 and pUL69 (Figure 4A, panels of lysate control) as well as their immunoprecipitates (Figure 4A, panels IP control) showed lower levels for deletion mutants ∆231–280 and ∆256–280 compared to WT and ∆231–255, thus reflecting the delay in replication kinetics (compare Figure 2). Interestingly, the two additional viral early proteins pp65 and pUL44 did not show this mutant-specific expression pattern. Quantitative values of band intensities were determined by densitometry of the Wb filters, defining the WT level as 100% and using this factor of normalization for the relative calculation of protein levels. Phosphorylation-specific bands (Figure 4A, panel IVKA ATPγS) were verified by a restaining of the IVKA Wb filters using monospecific antibodies as indicated. Notably, the pUL97 autophosphorylation as well as the phosphorylation of all three analyzed viral substrates showed a strong reduction for the largest deletion ∆231–280, a partial reduction for ∆256–280 and almost no reduction versus WT for ∆231–255 (Figure 4A, lanes 5, 4, 3 and 2, respectively). For mutant ∆231–280, the normalized levels of reduced phosphorylation signals were down to 9% or 21% for the two IP proteins pUL97 and pUL69, and 56% or 37% for the two CoIP proteins pp65 and pUL44, respectively. This mutant-specific reduction is considered a combined effect of two parameters, namely reduced phosphorylation, on the one hand, and reduced abundance of the proteins in the kinase complexes, as illustrated by the descending presence of protein bands in the IVKA Wb restaining (Figure 4A, right panels).

As a second readout of phosphorylation patterns, the Phos-tag system of Wb detection was used, which reflects the occurrence of various phosphorylated forms of each individual protein (Figure 4B, left panels). A subtractive phosphorylated/nonphosphorylated analysis was achieved by the treatment of cellular lysates with λ-phosphatase prior to loading on SDS-PAGE for Wb detection (λ-PP; Figure 4B, right panels). The quantitation of the Phos-tag Wb bands was achieved by densitometry, in that the quotient of phosphorylated forms (upper three bands) towards the total protein amounts was determined for each virus. This procedure confirmed, mostly for the two deletion mutants, ∆231–280 and ∆256–280, the reduced levels of pUL97 autophosphorylation (68% ∆231–280, 81% ∆256–280, 93% ∆231–255, 92% HCMV WT; Figure 4B, lane 5), whereas pp65 phosphorylation appeared unaffected. In the case of pp65, the Phos-tag effect was clearly visible by the slower migration pattern (compare with MW markers in Figure 4A), and, due to the massive hyperphosphorylation of pp65, the λ-phosphatase reaction retained some residual faint bands, but no reduced phosphorylation levels were observed for the mutants. This difference in findings for pp65 shown in Figure 4A,B may result from a predominant defect of the deletions on the interaction pp65-pUL97, but a much lower effect on pUL97-mediated pp65 phosphorylation. An alternative explanation may be given by the possibility of additional pp65 phosphorylation through pUL97-independent kinases [22,56,57,58], which is less detectable by CoIP-based IVKA than by Phos-tag analysis. Three further viral early proteins, pUL69, pUL50 and pUL44, showed a modest reduction in phosphorylation signals, which again appeared mainly noteworthy for the mutant ∆231–280, since this refers to the possibility of a multifactorial basis of the replication defect (compare Figure 2). As far as the phosphorylation of cellular proteins was concerned, an upregulation was seen for specific phosphorylated varieties of retinoblastoma protein (Rb), nuclear lamin A/C (note asterisk) and cyclin B1, all representing known substrates of pUL97 activity [16]. Interestingly, a reduction in phosphorylation in these cellular proteins in correlation with the UL97 mutation was poorly observed, when considering the relatively strong effects seen for viral proteins. This points to the possibility that the overall catalytic activity of pUL97 is not fully destroyed through the deletions, but that phosphorylation defects may occur in a substrate-specific fashion. Notably, one distinct change in the qualitative pattern of Rb phosphorylation was noted for the mutant ∆256–280 (Figure 4B, panel of mAb-Rb, lane 4). The occurrence of an unusual phospho-specific band might indicate altered coverage of alternative phosphosites in Rb.

Thirdly, distinct phosphosites in these cellular proteins were analyzed by the use of phospho-specific antibodies (Figure 4C). While most sites investigated were found largely unaltered in the case of the three viral mutants (Rb pSer807/811, Rb pSer608, lamin A/C pSer22 and cyclin H pThr315), prominent alterations were yet seen for two phosphosites in Rb. For Rb pThr373, a stepwise reduction in the quantity of phosphorylation was determined for mutants ∆231–255 (49%; lane 3), ∆256–280 (36%; lane 4) and ∆231–280 (9%; lane 5) compared to WT. Site-specific Rb phosphorylation regulates cell cycle progression [59] and is subject to pUL97-mediated regulatory phosphorylation through pUL97 in HCMV-infected cells [11], so that this phenotypical, fine-regulatory change may directly correlate with the reduction in viral replication capacity measured for the three deletion mutants. Additionally, Rb site pSer796 indicated a slight reduction for the mutant ∆231–280 (lane 5), thus underlining the distinct variations between this mutant and WT.

### 3.5. Confocal Microscopic Imaging of Intranuclear Patterns of HCMV pUL97–Cyclin Colocalization

**Figure 5:**
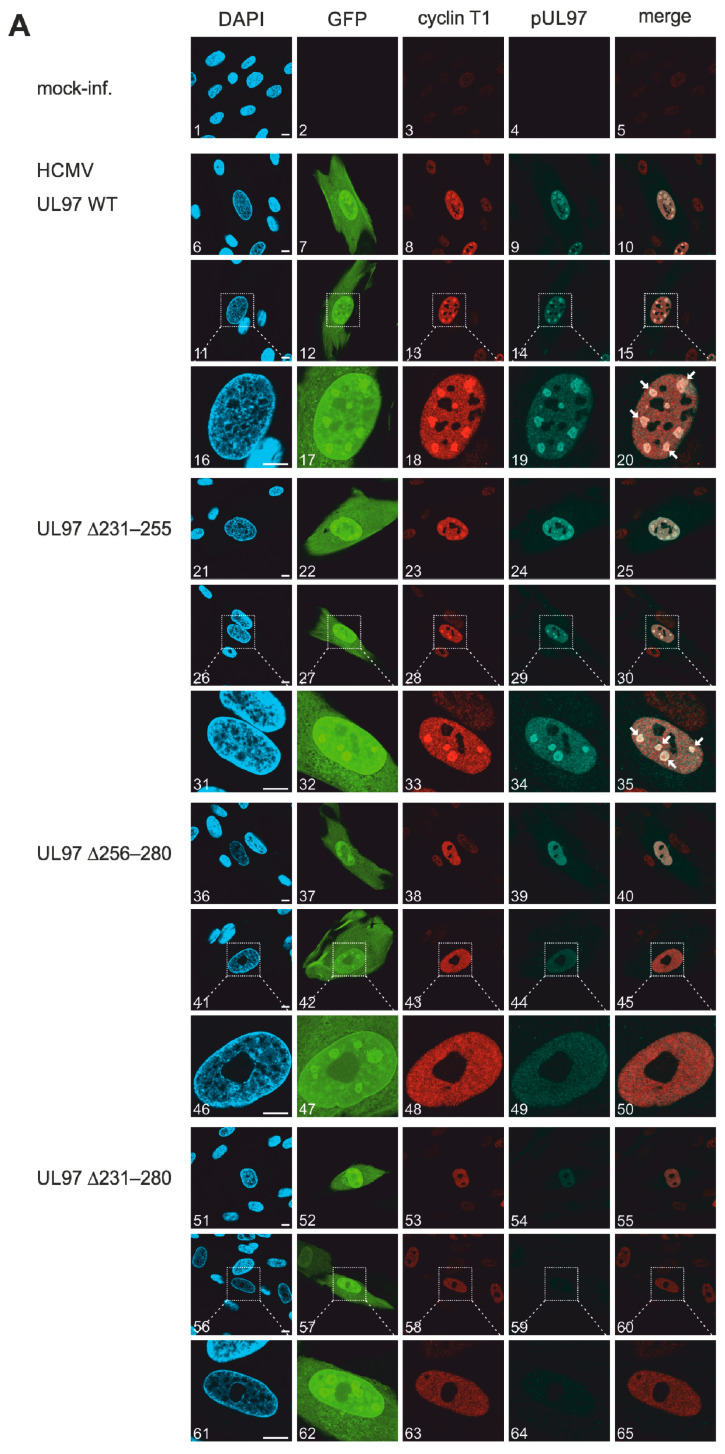

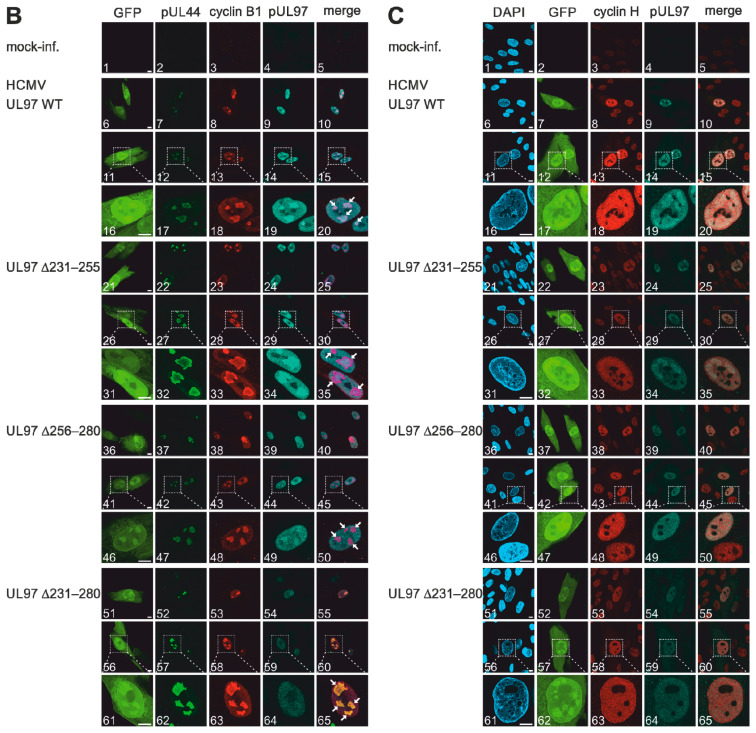
Confocal imaging analysis of the localization of human cyclins and UL97 mutants in HCMV-infected HFFs. HFFs were infected with WT and recombinant HCMVs at an MOI of 0.1 and harvested at 3 d.p.i. (marked by the GFP reporter expression of the recombinant viruses). Indirect immunofluorescence staining was performed with antibodies against pUL97 and human cyclins T1 (**A**), cyclin H (**B**) and cyclin B1 (**C**) and representative panels of confocal imaging are given. Regarding cyclin T1 (**A**) and H (**C**) stainings, secondary Abs anti-rabbit Alexa 555 was used to visualize human cyclins and anti-mouse Alexa 647 to visualize pUL97. Concerning cyclin B1 (**B**), pUL44 was stained to depict viral replication centers (marked by white arrows). pUL44 was visualized using anti-mouse Alexa 350, cyclin B1 by using anti-goat Alexa 555 and pUL97 by using anti-rabbit Alexa 647. Scale bar, 10 µm.

Our previous investigations identified a distinct colocalization of pUL97, and likewise substrates of pUL97, such as pUL69 and pUL44, with cyclins in viral nuclear replication centers [9,27,28,60]. These patterns of colocalization correlated with the pUL97–cyclin interaction identified by a number of different methods [16] and could partly be modulated by the treatment with pUL97- or CDK-inhibitory small molecules [27,61,62]. Here, the impact of UL97 deletions on colocalization with cyclins T1, H and B1 was analyzed by indirect immunofluorescence multiple stainings and confocal imaging (Figure 5A–C). Using HCMV-infected HFFs again as the cultured cell model, mutants ∆231–280 and ∆256–280 showed the most striking effect. While a strong speckled colocalization of HCMV WT and minor deletion mutant ∆231–255 with cyclins T1 and B1 was detected in viral nuclear replication centers (Figure 5A, images 18–19 and 33–34), the mutants ∆231–280 and ∆256–280 showed an impairment of cyclin T1 colocalization (Figure 5A, images 48–49 and 63–64). For both mutants, a smooth and non-speckled distribution of pUL97 was found throughout the nucleoplasm (excluding nucleoli), with no areas of speckled accumulation, and cyclin T1 was not recruited to such sites. This finding was consistent with the CoIP data showing reduced or lost cyclin T1 binding of ∆256–280 or ∆231–280, respectively (Figure 3). The picture was different for cyclin B1, as all three deletion mutants showed a preserved colocalization of cyclin B1 in viral replication centers, together with pUL44 (used for costaining as a marker of replication centers) and partly with pUL97 (Figure 5B, images 7–9, 17–19, 22–24, 32–34, 37–39, 47–49, 52–54 and 62–64). No difference was seen between the three deletion mutants and WT, which is in line with the CoIP data indicating the preservation of cyclin B1 interaction capacity. For cyclin H, smooth nuclear staining patterns were obtained without visible differences between the viral mutants (Figure 5C). For WT and ∆231–255, the presence of viral nuclear replication centers is recognized, at least in part, by the pattern of pUL97 (Figure 5C, images 19 and 34), but no centers or areas of speckled accumulation could be detected for cyclin H. Here, a loss of cyclin H interaction in mutant pUL97 could not be verified using confocal imaging (Figure 3). This was different to our earlier investigations, in which pUL97–cyclin H colocalization within replication centers was detectable under WT conditions, and this might be explained by the use of different anti-cyclin H antibodies in the separate experiments (limitation in continuous antibody availability). A quantitative evaluation of colocalization phenotypes was achieved by visual microscopic counting. The data specifically highlighted the impairment of cyclin T1 colocalization within viral nuclear replication centers for the three mutants ∆231–255 (78 ± 1%), ∆256–280 (11 ± 2%) and ∆231–280 (15 ± 2%) (Table 1). Thus, the results underline the occurrence of gradual phenotypical defects for these mutant HCMVs.

### 3.6. Measurement of pUL97-Specific Drug Sensitivity of the Viral UL97 Mutants

**Figure 6:**
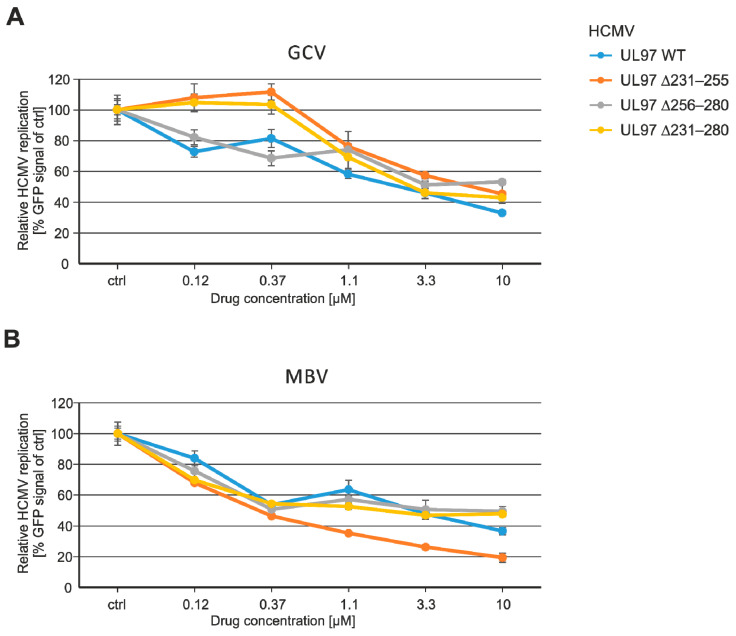
HCMV GFP-based antiviral assay was performed with the UL97 mutants in HCMV-infected HFFs (MOI of 0.25 GFP-FU/cell) to assess viral drug sensitivity (**A**,**B**). Mean values ± SEM of quadruplicate determinations are given (infections in duplicate, GFP measurements in duplicate).

In the next step, the drug sensitivity of recombinant HCMVs was assessed, using the established HCMV GFP-based replication assay, with a particular focus on the two pUL97-relevant antiviral drugs, GCV and MBV. For both drugs, a concentration-dependent profile of antiviral activity was measured for all four viruses analyzed. No general difference in the profiles of drug sensitivity was noted for the UL97 deletion mutants (Figure 6); only the deepness of the curve shapes showed some variation. A calculation of EC_50_ values indicated quantitative differences that remained within a factor of <2 (GCV) or <5 (MBV) compared to WT. Mutants ∆256–280 and ∆231–280 showed some decreased GCV sensitivity (increase in EC_50_ to 5.2 ± 0.9 µM or 4.4 ± 03 µM, respectively), while mutants ∆231–255 and ∆256–280 showed some increased MBV sensitivity (decrease in EC_50_ to 0.5 ± 0.1 µM or 1.8 ± 0.5 µM, respectively) (Table 2). None of the mutants acquired drug resistance, but the measured variations may point to some relative changes in the efficiency of drug–target binding. This appears noteworthy considering the fact that none of the small deletions are located within the GCV or MBV binding/resistance regions, i.e., amino acids 337–607 [16]. However, as a general limitation of these analyses, any introduced deletion may lead to a slightly altered protein conformation that might possibly also have an effect on drug accessibility. In this context, pUL97–cyclin binding is expected to induce specific conformational alterations, which may influence the catalytic domain and substrate recognition as well as drug binding properties. Interestingly, GCV or MBV are characterized by profound mechanistic differences, in that MBV is a classical inhibitor of the ATP binding site, whereas GCV is a prodrug that requires the activation of phosphorylation through pUL97 to develop antiviral activity. Due to these differences, the two drugs express an antagonistic effect upon cotreatment [63]. Irrespective of these mechanistic differences, both drugs share the necessity of proper access to their binding sites in pUL97 [64], so that the present data may support the idea that deleted regions might have a certain impact on these binding characteristics. However, the deletion mutant that showed a loss of cyclin T1 and H interaction, ∆231–280, did not acquire substantial changes in drug sensitivity compared to WT (factor of differences in EC_50_ for GCV and MBV both <2, low or no statistical significance, respectively; Table 2). Thus, mutations in this region of pUL97 did not indicate a clear correlation between cyclin binding and drug sensitivity.

### 3.7. Refined Model of the pUL97–Cyclin Interaction Based on the Combined Experimental Data of This Study

**Figure 7:**
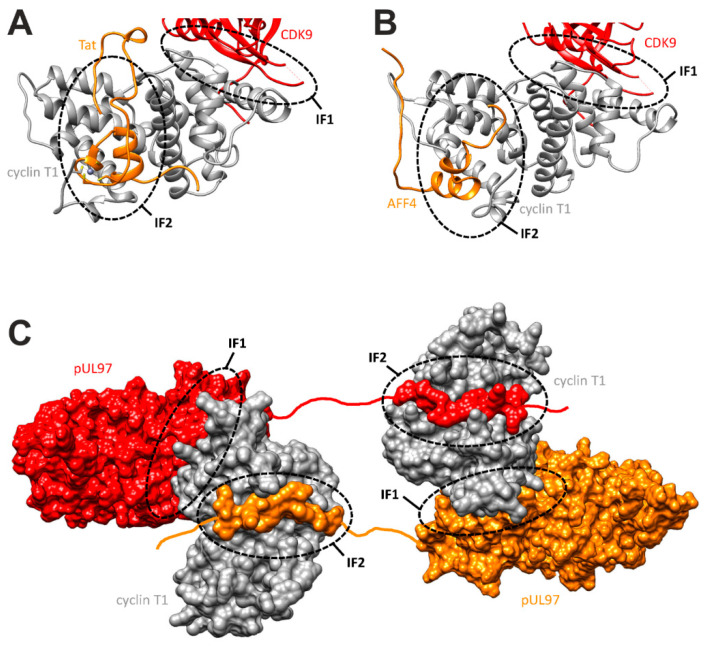
Cyclin T1 interaction sites and model of the cyclin T1–pUL97 complex. (**A**) Crystal structure of cyclin T1 (gray) with CDK9 (red) and Tat (orange). The two interfaces (IF1, IF2) used for these interactions are highlighted by dashed lines. (**B**) Crystal structure of cyclin T1 (gray) with CDK9 (red) and AFF4 (orange). (**C**) Model of a quaternary complex consisting of two pUL97 molecules (red, orange) bridged by two cyclin T1 domains (gray). pUL97(231–280) was modeled in an extended conformation in interface 2, whereas the globular pUL97 kinase domain was modeled to interface 1. Red and orange lines denote parts of the pUL97 structure for which no structural information is available.

The experimental data above show the importance of the sequence stretch 231–280 of pUL97 for cyclin T1 binding. To understand the molecular principles of this interaction in more detail, we have analyzed the structures of known cyclin T1 complexes with various binding partners. This analysis illustrates that cyclin T1 exhibits at least two distinct surface regions that are involved in protein–protein interactions. One of these interfaces normally mediates the contacts with the globular domain of CDK9, interface 1 (IF1), whereas a second, less well-defined surface region, interface 2 (IF2), is able to bind to short sequence segments of regulatory proteins, such as the Tat protein of human immunodeficiency virus type 1 (HIV-1) or the cellular factor AFF4 (Figure 7A,B). CDK9 that binds via IF1 shares sequence homology with the pUL97 kinase domain (amino acids 328–655). Based on this homology, we have previously modeled the cyclin T1–pUL97(328–655) complex [29]. pUL97(231–280) resembles the known ligands of IF2, namely Tat and AFF4, both with respect to the length and the absence of a globular domain structure in the respective region [28]. In addition, the presence of several cysteines and histidines in pUL97(231–280) suggests that this protein might contain a zinc finger, similar to the respective region of the Tat protein (Figure 7A). These similarities render it likely that pUL97(231–280) binds to IF2, although the role of individual amino acids and the exact binding mode still need to be determined. Combined, these considerations suggest that pUL97(231–280) and pUL97(328–655) interact with cyclin T1 via two distinct interfaces. This conclusion allowed us to extend our previous model [29] by the cyclin T1–pUL97(231-280) interaction (Figure 7C). In this refined model, the presence of two interfaces also offers a structural explanation for the cyclin-mediated pUL97 self-interaction via the formation of higher-order complexes, e.g., via a tetramer formed by two pUL97–cyclin T1 heterodimers (Figure 7C). Given this heterotetrameric model, which may explain the overlap in our previous CoIP-based mapping of pUL97–cyclin T1 and pUL97–pUL97 interaction activities, the IF2-mediated interaction obviously exerts a dominant or stabilizing role over the IF1-mediated interaction. This is concluded from the finding that deletion of IF2 in the mutant ∆231–280 (both in the recombinant viruses and in the transient plasmid expression systems) led not only to a loss of pUL97–pUL97 oligomerization but also to a loss of a detectable level of pUL97–cyclin T1 interaction. Thus, IF1 alone appears not to be sufficient for mediating a stable pUL97–cyclin T1 interaction.

## 4. Discussion

In the present study, we focused on the investigation of newly generated recombinant HCMVs that carry ORF-UL97 deletion mutations in order to address the question of whether phenotypical defects in cyclin interaction, kinase activity and/or viral replication may arise. The combined findings emphasize a number of interesting points. First of all, the replication of the three recombinant HCMVs, i.e., UL97 ∆231–255, ∆256–280 and ∆231–280, was impaired in the course of infection kinetics and overall replication efficiency (viral genome-based qPCR), seen mostly for the largest deletion, ∆231–280. This pointed to the functional importance of this region of pUL97, which is located in its nonstructured, N-terminal portion. Considering the fact that previous data, derived from transient expression systems, highlighted this region as a determinant of pUL97–pUL97 oligomerization, cyclin T1 interaction, autophosphorylation and, thus, putatively an activated state of the pUL97 kinase activity in general, the applied system with recombinant virus infections provided confirmation of this scenario.

It should be mentioned that a number of earlier reports described the phenotypical defects of HCMV replication under conditions of pUL97-directed drug treatment [65] (reviewed in [16]) or deletion of the entire ORF-UL97 from the viral genome [54,55]. Interestingly, the importance of pUL97 activity was specifically illustrated by comparing HCMV replication under maribavir (MBV) treatment only, or in combination with inhibitors of cellular kinases [66]. Here, the dimension of a viral replication defect was more drastic under combination treatment, thus demonstrating that some cellular kinase inhibitors enhanced the antiviral activity of MBV. Notably, our recent finding contributed to this notion, in that we identified a pronounced synergism of combination treatments with MBV and the CDK7-specific preclinical drug LDC4297, as quantitatively assessed by two established drug combination assays [63]. This may refer to the crosstalk and functional complementation between active cellular CDK–cyclin complexes and the viral CDK ortholog pUL97. In none of these studies, however, was the direct involvement of pUL97–cyclin binding in the regulation of kinase activity and viral replication specifically taken into account. Thus, to the best of our knowledge, this is the first study providing evidence for a correlation between pUL97–cyclin interaction and regulatory importance as seen with the phenotypical defects of the UL97 deletion mutants.

Due to the fact that the deletion-specific region ∆231–280 is responsible for both pUL97–cyclin T1 interaction and pUL97–pUL97 self-interaction/oligomerization (a prerequisite for pUL97 autophosphorylation and for attaining its active state), the present results, obtained with HCMVs carrying either full or partial deletion of this central region, demonstrate a gradual degree of replicative defects for ∆231–280 (major defect) compared to ∆231–255 and ∆256–280 (minor defects). The detailed characteristics of these defects are addressed in Figure 2, Figure 3, Figure 4, Figure 5, Figure 6 and Figure 7. In an earlier study, the attempts to generate simple amino acid replacement mutants of pUL97 with the aim of producing a loss of cyclin interaction while retaining basal kinase activity in vitro largely failed. Particularly for pUL97–cyclin B1 interaction, all approaches to site-specific mutagenesis failed to identify a cyclin B1-negative but kinase-positive pUL97 mutant, thereby leading to the conclusion that pUL97 kinase activity is essential for the cyclin B1 interaction [9]. This correlation appeared to be different for the case of pUL97–cyclin T1 and pUL97–cyclin H interaction, for which pUL97 kinase activity was not found to be a strict requirement, specifically supported by the present report. In this context, it is interesting to note that even the existence of ternary complexes pUL97–cyclin-T1–CDK7 could be demonstrated experimentally by an approach involving protein in vitro assembly [9].

A new finding of the present study was that the viral mutant ∆231–280 showed a loss of interaction with cyclin T1 and cyclin H (CoIP and confocal colocalization), but still showed interaction with cyclin B1. This pointed to the importance of cyclin T1 and cyclin H interaction for viral replication, at least in vitro in primary human fibroblasts, even when pUL97–cyclin B1 interaction was still functional. It is possible that cyclin B1, which requires active pUL97 for interaction, regularly undergoes pUL97-specific phosphorylation (similar to CDK-specific cyclin phosphorylation), whereas cyclins H and T1, both showing independence of pUL97 activity for interaction, may not serve as pUL97 substrates but may exert phosphorylation-independent regulatory effects during viral replication. Moreover, the kinase phenotype of ∆231–280 indicated a strong impairment of both autophosphorylation and substrate phosphorylation activities (IVKA, Phos-tag and phospho-specific antibody stainings). The role of autophosphorylation in pUL97 catalytic activity has been a point of controversial discussion [9,34,67,68], but the present data strongly argue for our earlier statement that autophosphorylation is linked to oligomerization, and that these combined events may represent codeterminants for achieving the kinase’s active state. None of the deletion viruses showed resistance against GCV or MBV, so that the discrimination between drug binding regions (337–607) [16] and this functionally important region of pUL97 oligomerization/cyclin T1 interaction (231–280) could be confirmed. Finally, a bioinformatic assessment of the pUL97–cyclin T1 complex led to a refined model, suggesting a tetramer formed by two pUL97–cyclin T1 heterodimers, thus expanding our understanding of this key-point of virus–host protein interaction.

Notably, an earlier study by Wolf et al. (2001) described the variety of phenotypical changes arising from an entire deletion of the ORF-UL97, i.e., HCMV ∆UL97 [54,55], such as reduced levels of viral gene expression, delayed genomic replication and, most importantly, impaired nuclear capsid egress, but not the complete replication incompetence of this ∆UL97 mutant. In the present study, the situation with the HCMV UL97 ∆231–280 mutant was found to be very similar. Moreover, here, no single, clear-cut replicative defect could be defined, but, instead, several functionally linked pUL97-specific impairments were identified. These comprised the lack of a quaternary pUL97–cyclin T1 complex, which is considered a prerequisite for pUL97 oligomerization and full activity, as well as the reduced protein levels of pUL97 and substrates, reduced pUL97–substrate interaction signals and defects in pUL97-mediated phosphorylation. Consequently, the replication kinetics of this mutant were delayed and quantitatively reduced. On the basis of these data, the cyclin T1 binding region of pUL97, 231–280, proved to be an important functional determinant of viral replicative fitness. Further studies should address additional mechanistic aspects of pUL97–cyclin interaction, also illustrating its putative impact on the recognition, phosphorylation and functional modulation of pUL97 substrates.

## Figures and Tables

**Table 1:** Colocalization of cyclins in viral nuclear replication centers *.

HCMV	Cyclin T1	Cyclin B1	Cyclin H
UL97 WT	92 ± 1%	>95%	<5%
UL97 ∆231–255	78 ± 7%	>95%	<5%
UL97 ∆256–280	11 ± 2%	>95%	<5%
UL97 ∆231–280	15 ± 2%	>95%	<5%

* Quantitation was performed by visual microscopic counting. HCMV pUL44 was used as a marker of viral nuclear replication centers and human cyclins were co-stained using specific Abs. Here, 15–20 areas containing 5–15 HCMV-positive cells were counted in duplicate and mean values ± standard error are given.

**Table 2:** EC_50_ values that demonstrate the antiviral in vitro efficacy of ganciclovir and maribavir against recombinant HCMVs *.

HCMV	EC_50_ Ganciclovir (µM)	EC_50_ Maribavir (µM)
UL97 WT	2.6 ± 0.2	2.5 ± 0.2
UL97 ∆231–255	2.6 ± 0.8 (n.s.)	0.5 ± 0.0 (****)
UL97 ∆256–280	5.2 ± 0.4 (**)	1.8 ± 0.2 (n.s.)
UL97 ∆231–280	4.4 ± 0.2 (*)	2.7 ± 0.3 (n.s.)

* Raw data for the calculation of EC_50_ values are derived from Figure 6. Mean values ± SEM of quadruplicate determinations are given. Statistical analysis was performed using ordinary one-way ANOVA and Dunnett’s test compared to HCMV UL97 WT. ****, *p* < 0.0001; **, *p* < 0.01; *, *p* < 0.05; n.s., not significant.

## Data Availability

The responsible authors declare that this article fully complies with the Data Availability Statements in section “MDPI Research Data Policies” at https://www.mdpi.com/ethics, accessed on 21 June 2021.
